# The Hospital. Nursing Section

**Published:** 1907-01-26

**Authors:** 


### The Hospital.

### Nursing Section.

Contributions for "THE HOSPITAL," should be addressed to the EDITOR, "THE HOSPITAL" NURSING SECTION, 28 & 29 Southampton Street, Strand, London, W.C.

No. 1,063.—VOL. XLI SATURDAY, JANUARY 26, 1907.

### Notes on News from the Nursing World.

### RESIGNATIONS AT DARLINGTON HOSPITAL.

WE understand that Miss F. A. Hunt, lady superintendent of Darlington Hospital, has resigned, and that other members of the nursing staff are also relinquishing their posts. It is just over 20 years ago since Miss Hunt became matron of the institution which she is leaving in about a couple of months. She was trained at St. Thomas's Hospital, and afterwards was for a time matron and secretary of the Hospital for Women in Marylebone Road. When she went from London to Darlington she did not expect to remain there for more than a short period, but she found the work so congenial that she stayed on, and under her auspices servants' quarters, the porter's lodge, and the children's ward were added in succession. Quite recently the important extension embracing two wards containing 14 beds, two rooms for single patients, a large laundry, a mortuary, a new operating-room, a new sitting-room and other accommodation for the nurses, has been completed. Before these additions were made there was so much pressure with regard to space that nurses were occasionally obliged to sleep in the committee-room. Miss Hunt believes that nurses ought to acquire a knowledge of hospital construction, and during the creation of the extension she did her best to explain to the members of the staff the principles on which it was being built.

### A MATRON ON MATERNITY TRAINING.

THOSE who are disposed to despise the calling of monthly nurses or to minimise the importance of proved capacity and sterling character in a trained midwife should read the remarks of the matron of the British Lying-in Hospital to our Commissioner in the report which appears on another page. Miss Knott expresses her conviction that no one ought to take up maternity work who does not possess an inherent love for small babies and is not able to maintain her interest in them. She also insists upon the necessity of possessing great patience with them and great sympathy for them. She shares the view that it is better for a nurse to take general training before midwifery training, instead of after, because a fully-trained nurse has more confidence in tackling the emergencies which frequently arise in midwifery cases than an inexperienced young woman. The great increase in the number of applications for midwifery training at the British Lying-in Hospital from fully-trained nurses is a hopeful sign of the times.

### NURSING AT THE ITALIAN HOSPITAL.

THE Governors of the Italian Hospital, who held their annual meeting last week, affirm that the cost per occupied bed compares favourably with any other hospital in London. This is largely due to the self-denying labours of the small band of sisters who undertake the nursing. The sisters belong to the community of St. Paul, and, including the Sister Superior, are ten in number. Two are engaged in the pharmacy, one is in the linen-room, and one is practically housekeeper. There are in addition four nurses, three fully-trained and one half-trained; two of these perform night duty work, one sister—who has already been on duty during the day—staying up until 12 or 1 o'clock or all night if necessary. Besides the nursing staff, there are wardmaids and servants for the kitchen. The accommodation for the nurses is of a somewhat makeshift character; the two night nurses have a room outside the hospital, and there is a small room on the ground floor, which is used as a sitting-room. Although preference is given to Italians, as many as 6,000 British subjects made use of the hospital during last year. The wards are all airy, bright-looking rooms, kept absolutely speckless. There are several single bed wards, which are found very convenient for certain patients. A glance into the out-patients' hall and the pharmacy showed two sisters hard at work dispensing and a good array of patients waiting their turn.

### SCANDALOUS NEGLECT AT A YORKSHIRE INFIRMARY.

A VERY alarming incident occurred the other day at Dewsbury Workhouse Infirmary. At the conclusion of an entertainment in the wards, the matron, Miss Payne, asked the Rev. Edwin Carr, who had been conducting it, to assist her in putting the strait-jacket on a patient who was obviously out of his mind. Father Carr complied, and while they were trying to put the jacket on, the man escaped, ran to his locker, drew a table-knife from the drawer, and attacked the priest. A struggle ensued, and the patient, still in possession of the knife, rushed from the ward, slammed the door behind him, struck at the porter with the knife, and getting outside the infirmary, was only captured with difficulty by a policeman. Mr. Carr, in commenting on the incident, impeaches the management of the infirmary, and avers that "cases of mental debility of every kind are treated in the ordinary wards, where the sick inmates are," also that "mental patients have been known to hide knives under their pillows and elsewhere." There are no male officials in the building, which is isolated and stands apart. An experienced adminstrator will realise the danger if the acute mania had seized the man when only the two night nurses, each on a separate floor, were on duty. Indeed, Mr. Carr states that "less than twelve months ago a man suddenly went mad in the night, pinned a night nurse by the throat in the corridor, and injured as well as terribly frightened her before her colleague came to her assistance." The Guardians had therefore knowledge of the danger, and they are consequently guilty of scandalous neglect in not affording proper protection to the nurses at Dewsbury Infirmary.

### NURSING IN NEW ZEALAND TWENTY YEARS AGO.

LAST week we mentioned the difficulties under which New Zealand nurses have to train, and congratulated the authorities of some of the leading hospitals on the results they have none the less been able to achieve. On another page to-day a contributor, who was in the Dunedin Hospital in 1886, gives a vivid account of her experience when she entered that institution as the first fully-trained nurse engaged in the wards. We fancy that the modern nurse would say that the work she had to get through was an impossibility. But the fact that she remained in the hospital for two years and not only nursed 22 patients from 6 A.M. to 6 P.M., but polished the brasses, scrubbed the ward kitchen, tables, and bath-room every day, and the whole ward once a week; cleaned her own fireplace after carrying up her logs from the basement to feed it, folded all the ward linen, and occasionally ironed it, certainly proves that she was equal to any demands upon her. In those days a female nurse never went into a male ward, and our contributor, who suggested that she had no objection to do so, was looked upon by the untrained matron as hardly respectable.

### SLIGHTING THE SUPERINTENDENT NURSE.

A SUPERINTENDENT nurse of an important poor-law institution, writing to us to express her gratification at the plain speaking of our contributor "M.D." on workhouse nursing, gives a remarkable instance of the imperative need of reform in the existing system. She states that not only is she in total ignorance of the credentials of a nurse when she is appointed, but that she does not have an opportunity of even speaking to her "until she comes to commence her duties." Could anything be more ridiculous than this? Apart from the slight which is put upon the superintendent nurse, what can be said in defence of the policy of allowing nurses to be engaged without consulting the one person in the institution who is qualified to ascertain whether they are fit for the work?

### FADDISM AT SAN FRANCISCO.

THE authorities of the City and County Hospital, San Francisco, lately made it a rule that the nurses must habitually wear white shoes and stockings. The innovation caused much irritation among the staff, and a remonstrance with many signatures was drawn up and presented to the principal of the training school. For a time the new rule was disobeyed. This resulted in an order on the part of the Board of Health—the governing body—that any nurse appearing in black shoes and stockings would not receive a pass to leave the grounds, and all but two of the staff gave in. They, it is stated, still wear black shoes and stockings, and go without the pass. Resistance to rules cannot be countenanced, but it reads rather as if there were faddists in power at the San Francisco Hospital.

### MILK-AND-WATER REFORMS AT HOPE HOSPITAL.

ON Thursday last week the Infirmary Committee of the Salford Board of Guardians met in order to discuss the report of the Inspector of the Local Government Board on the administration of Hope Hospital, to which we alluded last week. It was determined to call the attention of the steward and the matron to the matters complained of in the report, and "to warn them that if a similar state of things occurred again they would be dismissed forthwith." The committee also resolved to recommend the appointment of a senior resident medical officer "with modified duties" as medical superintendent, of a night porter, and a bath attendant. One of the committee, a lady, urged the importance of a revision of the dietary scale, and in particular the substitution of butter for margarine for the sick inmates, but a decision on this point was deferred to a future meeting. The committee evidently do not recognise the gravity of the situation, and we doubt if anything short of drastic action on the part of the Local Government Board will induce the Salford Guardians to realise and discharge their duty.

### INSUBORDINATION AT WOLVERHAMPTON.

THE Wolverhampton Guardians have dismissed a probationer for a serious breach of the regulations. Having first left a ward of sick people without anyone in charge, she subsequently, on being remonstrated with, obtained a cab and quitted the institution. The chairman, in criticising her conduct at the meeting of the Board last week, insisted that it was requisite, in the interests of discipline, to dismiss her, instead of accepting the resignation which she had sent in, and also to deprive her of the amount of salary due. We think that the Guardians were fully justified in assenting to this proposal. That the rules at Wolverhampton are not generally felt to be harsh is shown by the fact that after the discussion on the case of insubordination, a testimonial for efficiency was presented by the chairman to another nurse whom he complimented upon her work and success.

### NURSES AND MONEY-LENDERS.

A CLAIM was made the other day by a firm of money-lenders at Darlington against a nurse in the Bootham Mental Asylum at York, for £16, money lent under a promissory note. The loan was for £10, and the defendant, in her evidence, said that she spent part of it on clothing and part on a holiday at Leeds. In cross-examination she stated that she had borrowed £10 from money-lenders before and had repaid it. An order for repayment was made in this case, £3 forthwith and the remainder per £1 a month, with costs. We hope that the experience of the nurse in this case will be a warning to others not to apply for financial help to money-lenders. Nurses, like other people, ought to arrange their expenditure on dress according to their means, and however much a holiday may be needed, the expedient of borrowing money at ruinous interest in order to obtain it, is certain to end in disaster.

### NO NIGHT NURSE AT NORTHALLERTON.

The Northallerton Guardians have resolved, in face of the warning of their medical officer, not to have a night nurse for the workhouse infirmary. There is no question as to the grave view taken by the medical officer. He concludes his letter to the Chairman by saying, "To leave these poor people unattended during the long hours of the night in an infirmary provided and managed by the Guardians of the poor, would be grievous neglect, and it shall not be done without an official protest by me." It has been agreed that "if a serious case arises" a night nurse is to be called in. Such a decision shows little heart and less knowledge on the part of those who sanctioned so crude and wanton a proposal. Will not the ratepayers of Northallerton join their formal protest to that of the medical officer against this neglect of the sick poor on the part of their Guardians?

### AN INCIDENT AT BRISTOL.

At Bristol Police Court last week a certificated nurse, aged 36, was charged with publishing a scandalous libel on a well-known citizen, and also with attempting to commit suicide by throwing herself over Clifton Suspension Bridge. Both charges were proved, and the accused, having made a complete apology to the gentleman she libelled, was bound over to be of good behaviour for six months. The interest of this case to us lies in the fact that the convicted person has been, until recently, matron of a maternity home. The evidence given at the police court tends to justify the conclusion that if the registration of nursing homes were compulsory she would not have had the opportunity of filling a position in which such qualities as self-control and self-respect are indispensable.

### EPILEPTICS AND LUNATICS.

In consequence of certain statements having been made respecting the treatment of patients in the imbecile ward of Leigh Workhouse, the Clerk to the Guardians replied at length to them at a meeting of the Board. We are not concerned with the precise matters on which the controversy arose, but we notice that the Clerk admitted that some epileptics are placed with lunatics, and pleaded that the same plan is pursued "in most of the Unions in this country." He also affirmed that it was the original intention, when the buildings were altered, that one portion should be set aside for the epileptics, but that it had not been found possible to carry out the intention. Nevertheless, no system of mental nursing can be deemed satisfactory in which provision is not made for the separation of epileptics and lunatics.

### VACANCIES IN THE MILITARY NURSING SERVICE.

The matron-in-chief of Queen Alexandra's Imperial Military Nursing Service may now be seen on both Tuesdays and Thursdays at the War Office, Whitehall, between the hours of 10 a.m. and 1 p.m., by candidates for admission. There are still vacancies for staff nurses, which will be filled at once; but the applications are numerous, and the most eligible candidates have, of course, the best chance of being selected. The conditions of service and rates of pay have so frequently been given in our columns that we need only add that applicants must be between the ages of 25 and 35, and must possess a certificate of not less than three years' training in medical and surgical nursing in a civil hospital of not fewer than 100 beds, and recognised by the Advisory Board.

### GUY'S HOSPITAL.

On Friday evening last the members of the Pavy Gymnasium—assisted by some professional and amateur outsiders—gave their annual display and ladies' night in the Physiological Theatre at Guy's Hospital. The chairman, Dr. Pavy, was present, and some of the medical staff. The nursing staff was also well represented. There were some brilliant feats of swordmanship and fencing and "the Handcuff King" gave an exhibition. There was also an "Indian club solo." The proceeds of the performance are given to the Convalescent Home and Children's Home at Honor Oak Park.

### LEGACY FOR THE JUNIUS S. MORGAN BENEVOLENT FUND.

By the will of the late Miss Charlotte May Bann, of 16 Church Road, Newton Abbot, the Junius S. Morgan Benevolent Fund receives a legacy of fifty pounds. This generous donor, who has so practically exhibited her sympathy with nurses, was a sister of Miss E. M. Bann, matron of the Brook Fever Hospital, Shooter's Hill.

### MISS LOW'S CONDITION.

The English nurse attached to the Nurses' Co-operation, who was sent out by Mrs. Lucas to attend Miss Low, reports that she is making satisfactory progress. She has been transferred to a private room at the hospital in Chambery, and her friend, Mademoiselle Lechat, visits her constantly.

### SHORT ITEMS.

The first Pound Days have just been held at Malton, Norton, and District Cottage Hospital, and the result is very encouraging seeing that the building was only opened seventeen months ago. On the two Saturdays set apart, 957 pounds in kind were received and £3 7s. in cash.—We are officially informed that Miss E. Shackleford, who has recently been appointed matron of the Royal Portsmouth Hospital, had previously held the position of assistant matron there for three years and a half.—At a meeting this month of King Edward the Seventh's Coronation Fund for Nurses in Dublin, applications for membership from 13 nurses were considered and accepted.

### The Nursing Outlook.

"From magnanimity, all fears above;
From nobler recompense, above applause,
Which owes to man's short outlook all its charm."

### THE NURSE IN COUNTY COUNCIL SCHOOLS.

THE work of the visiting nurses in the County Council schools is still a quite new thing, but there is no doubt that its value is appreciated by those in authority. This is proved by the fact that last April the staff of nurses in London was increased from 12 to 32. At present these nurses work independently, but Sir Shirley Murphy, Medical Officer of Health for the County of London, states in his report that it will soon be necessary to appoint a superintendent of nurses, in order that the work may be properly controlled and kept at a uniform level of efficiency. Children, parents, and even teachers, may occasionally put difficulties in the way of the necessary investigations, which a nurse who is timid or inexperienced might find it troublesome to deal with, but which would vanish at once under the influence of a tactful senior officer. An influence, however, which will make teachers keener on the subject of cleanliness is the fact that His Majesty's Inspectors of Schools are now taking note of the condition of the children in their reports. In one case an inspector complained that in a certain school many of the children were so dirty, as to be offensive both to their companions and to the teachers. He added that "unless considerable improvement was made in this important matter, the school could not be regarded as satisfactory." Teachers and managers will recognise the real weight of these very mild-looking words, and a criticism like this will make them welcome the visits of the nurse.

The nurse's work begins with insisting on cleanliness, though it does not end there. One of her most constant duties is the examination of the children's heads. The school nurses examined, in all, the heads of 72,993 children during 1905, of whom 61,983 were proved to be clean. Of the remainder, who were verminous, the majority, indeed more than half of the total offenders, were senior girls—that is, girls beyond the infant department. Some of these, but proportionately not many, were cleansed after the nurse had noticed their condition, without formal notice to the parents. Many waited for the first warning, and a third were not cleansed until the red card which gives the final warning, had been sent. Even then, 963 of the original 6,723 offenders remained in a filthy condition, until the nurse proposed that they should be excluded from school, and of these 506 were excluded and the parents threatened with prosecution. Prosecutions of the parents of 101 children actually took place.

Next to the girls the children in infant departments were the most to be complained of, though in much smaller proportion. Probably in these departments also it was the girls who were most offensive. The parents of 101 children were prosecuted and fined, but the preventive work of the nurse, and the habits of cleanliness enforced and begun through her warnings, are of more importance than the prosecution of the almost hopelessly neglectful parents, who require to be brought into court and fined for carelessness in such an obvious duty.

While, however, the nurse's work in compelling parents to keep their children's heads clean has made great progress, only a beginning has yet been made in the equally important but much more difficult task of enforcing cleanliness of the body and the clothing. In the Borough of Marylebone, however, the nurse examined the children in seven schools and found 65 whose bodies and clothing were so verminous that they were unfit to be in school. The names and addresses of these were sent to the medical officer of health, whose sanitary inspectors delivered cards, explaining, that the children could have warm baths and have their clothes sterilised free of charge, on given dates, within certain specified hours. This offer was taken advantage of by 36 of the children, 28 were made clean by their mothers, and only one left the school. At present the baths are available only at disinfecting stations, and in one case at the casual ward of the workhouse. While this remains the case, it will be difficult to insist on a high standard of cleanliness among children, who have but little convenience for ablutions at home. If, in this country, as in Germany and Holland, there were baths attached to each school, personal cleanliness would be more easily attained, and therewith a higher standard of health among the children. As for clothing, one can but hope that, the damage done to fabrics by the sterilising process will incite mothers, who are at present careless, to cleanse their children's garments in the domestic wash-tub.

While these homely duties of looking for dirt and insisting on cleanliness remain the bulk of the nurse's work, there is also much important duty to be done in observing suspicious cases which may develop into infectious disease. The authorities of London are now asking for powers similar to those conferred on Glasgow by a Police Confirmation Act of 1904, by which it is ordained that, if a child is found by a medical officer or nurse to be in school in an objectionable condition, the parents may be ordered to cleanse the child within 24 hours, and if they fail to do so, the child may be cleansed at the public expense, and the cost recovered from the parents. If the right to do this were conferred on our school nurses, there is no doubt, they would be able to enforce the cleanliness desired with much less trouble and delay than at present. Hence it is to be hoped that the necessary powers will be granted ere long.

### Nursing in Tropical Climates.

By Andrew Duncan, M.D., B.S., M.R.C.P., F.R.C.S., Fellow King's College, Lecturer on Tropical Medicine at the London School of Tropical Medicine, and the Westminster Hospital.

### V. ENTERIC FEVER

(*Continued from page 219.*)

In concluding our remarks on diet, it may be mentioned that unless the patient is in a state of stupor he need not be awakened if sleeping. He may be allowed to drink freely in the intervals of his feeds of water. or of home-made, not bazaar-purchased, lemonade from which the pips have been strained.

Before proceeding to discuss the general nursing of the patient, I would direct attention to the especial dangers that are imminent over an enteric fever patient, namely hæmorrhage, perforation of the bowel and its consequent peritonitis, and cardiac collapse, which, in my experience, has been more frequent in cases in the tropics than in those in temperate climates.

As regards *hæmorrhage*, Murchison found it to occur in the largest number of cases in the third week, so that the nurse should bear this in mind. It will be shown to have occurred by a sudden increase of the prostration, pallor of the surface, increased frequency of the pulse, and a sudden fall in the temperature. If the hæmorrhage be large in amount it often brings on fatal collapse, or may be followed by perforation: if small, it is not of so much consequence. With the onset of these symptoms the nurse should at once apply ice to the abdomen, and the best place to apply it is over the right iliac region; a waterproof sponge-bag will make an efficient vehicle for the ice. The patient should be given ice to suck, and be kept perfectly still. Further directions will be given by the doctor, who should be at once summoned. It must be remembered that the blood may be retained in the bowel and not appear in the stools. Lastly, if there are signs of collapse, the foot of the bed should be raised.

*Perforation* will be indicated by the sudden onset of severe abdominal pain increased on pressure, quickening of the pulse, rapid lowering of the temperature, vomiting, and an anxious, altered appearance of the facies. Complete rest must, of course, be enjoined, the fæces and urine being directed to be passed into the draw-sheet, and not into the bed-pan, thus avoiding any movement. The bed-clothes should be kept off the patient by a cradle, band-box, or pillows. No food to be given by the mouth, but entirely by the rectum. Ice may, however, be sucked. The doctor should be sent for at once. This distressing accident occurs most frequently in the third, fourth, or fifth week of the disease.

*Peritonitis* may arise also without perforation. Here the doctor will order the necessary remedy; it may be mentioned that hot spongio-piline is more easily tolerated than poultices by the patient.

*Cardiac Collapse.*—This, as remarked above, is found more frequently in the tropics than in temperate climates. It is perhaps commoner in the third week. The respiration becomes quickened, is very distressing, and sighing in character. The impulse of the heart is very feeble and diffused, or may not be perceptible. The necessary directions will, of course, be given by the physician. I well remember a case of a brother officer who was nearly at the point of death from collapse, so much so that arrangements were made for his funeral in a few hours' time, it being in the hot weather. He was literally rescued from the jaws of death by the frequent administration of the subcutaneous injections of strychnine over the cardiac area.

Let us now consider the general nursing of the patient. Attention has already been drawn to the points relating to the room in which the patient lies and his feeding. First of all the enteric fever patient, it is needless to say, should be kept at perfect rest, this more especially in view of the dangers we have just been considering. The bed should not face the window, as this distresses him. He should never be allowed to raise himself for the purpose of getting anything from a table or chair by the bedside; but the nurse from time to time should carefully change his position in the bed, so that pressure be not constantly exercised on one part of his body, and thus occasion bed-sores. The occurrence of a bed-sore is generally a subject of reproach to the nurse. To obviate their appearance, the body must be kept scrupulously clean; it should be washed entirely night and morning, as this is most grateful to the patient. Any soiled bed-clothes will be removed at once. The mattress must be firm and elastic—a spring mattress with a horse-hair one over it is the best—and the bed-clothes kept free from creases and bread-crumbs. The skin must be ever kept completely dry, the draw-sheet should never be left wet, and this will have to be carefully looked after where there is incontinence of fæces and urine; in this latter case it is advantageous to anoint the skin with some greasy application such as lanoline or zinc ointment.

The usual sites for a bed-sore are the buttocks or hips, but they may occur anywhere. The skin in the above especial parts should be protected from the first day of the fever by rubbing it with—night and morning—spirit, such as brandy or eau de Cologne, or Friar's balsam, and then painting with collodion. The back must be examined once a day, and if any redness of the skin be discovered, then at once change the position of the patient, and let him lie on a circular pad or water-bed, and report the occurrence to the medical attendant. Should a bed-sore, in spite of all precaution, form, the nurse should keep the dressings ordered, on by the strapping, as bandages are apt to ruck up.

*The mouth and nose* should be looked after with care, as secretions are apt to dry and cake on them in hot climates especially. Hence, frequently during the day and night, and especially after food, swab out the mouth with some weak antiseptic lotion. The teeth must be brushed morning and evening. If the mouth is not regularly cleaned, inflammation may arise, spreading through the Eustachian tube to the ear or down the larynx. Look out for any excoriation on the lips and tongue, and, if present, report to the medical attendant. If the mouth is in a good condition the patient will take his food far more readily.

*The temperature* should be taken at four-hourly intervals. Any sudden rise or fall must be at once reported. But inasmuch as a continued high temperature is largely responsible for the wasting and exhaustion of the fever, many authorities give directions that, as soon as it reaches 103°, measures for reducing it should be adopted, such as sponging the body, or applying iced water compresses, frequently changed, to the body.

In America the cold-bath treatment is by many practised. Thus, Osler states that for many years it has been his practice to give a bath of 70° every third hour when the temperature was above 102.5°. The patient remained in the bath for fifteen to twenty minutes, after which he was taken out, wrapped in a dry sheet, and covered with a blanket. Whilst in the bath the limbs and trunk are rubbed thoroughly. Food is usually given, sometimes with a stimulant, after the bath. The mortality by this treatment certainly appears to have been lessened. Thus at Brisbane Hare reduced his mortality from 14.8 per cent. to 7.5 per cent.

As it is unusual to find a bath in the tropics in which a patient can lie at full length, mention may be made of an apparatus contrived for this purpose by Henderson, of Shanghai. This consists of a long bamboo settee, used by the natives of China, in which the cross-bar at the lower end is removed to make way for the bucket to catch the water as it runs away. Underneath the seat of open bamboo-work a receiver of some waterproof material is fastened by tapes tied along the side of the couch, and sloping towards the lower or bucket end. The water is then continually poured over the patient, who is stripped and covered with a sheet. This was invented by Henderson for the treatment of sunstroke, but can be used for the bath treatment of enteric equally well.

Another method is the "bed-bath." Around the edges of the bed are placed rolled blankets, and over these a rubber sheet, into which two or three pailfuls of water are poured. The patient is placed in the trough thus formed.

*Points related to the excreta.*—If there be more than four motions per diem the matter should be reported to the doctor, who will give his directions. If there be constipation this should also be brought to his notice, but on no account should the nurse give purgatives. The stools should, whenever passed, be examined for curds, in order to see if the milk be duly digested, or for sloughs. They should be covered over and saved for the doctor's inspection. Where there is incontinence of fæces pads of absorbent wool, sprinkled with some antiseptic powder and enclosed in butter-muslin, should be placed beneath the patient, and burnt immediately after removal. The method of disinfecting the stools will be described later on.

As regards the urine, there may be retention, which, of course, must be brought to the visiting physician's notice. After the catheter has been used, this must be scrupulously cleaned and sterilised. Where there is incontinence of urine this must be reported, and especial care taken as regards bed-sores, all soiled clothing being at once removed. The bed-pan must be used from the first, and care must be taken not to bruise the patient with it.

*Tympanites* is best met with by the application of the turpentine stupe or by the long rectal tube. Sir Wm. Jenner used to direct a flannel roller to be placed beneath the patient, then a double layer of thin flannel, wrung out in very hot water with a drachm of turpentine mixed with the water, was applied to the abdomen and covered with the ends of the roller.

*Epistaxis.*—This may be troublesome. When it appears the nurse should raise the patient's head on a pillow, and bathe his forehead and nose with ice-cold water. Should this not stay the bleeding, the doctor may have perhaps to plug the nostrils.

*Lung Complications.*—We usually find a certain amount of bronchitis in a case of enteric, and this may cause much sleeplessness from the constant cough. A more formidable complication is pneumonia. Where this occurs we must not allow the patient to be continually on his back, as by so doing the lower parts of the lung become much congested, and a hindrance to the necessary amount of air arises. Hence, the nurse should be directed to change the position of the patient in bed, first turning him on one side and then on the other, supporting him in the required position by pillows.

*Phlebitis* is more likely to complicate enteric fever in the tropics than in temperate climates. The veins of the lower extremity are most usually affected. When this occurs, the nurse will find the leg swollen and tender, these symptoms having appeared more or less suddenly. The nurse must impress on the patient on no account to move the parts, lest a portion of the clot be loosened from the vein into the circulation, when sudden death might occur. The limb should be elevated and bandaged carefully, beginning at the toes.

*Tender toes.*—Handford describes a curious condition of the toes, which become extremely tender at their tips and pads, so that the weight of the bed-clothes cannot be borne. It has been found most common after the bath treatment. The nurse should take off the weight of the bed-clothes here by a cradle.

*Mental symptoms.*—There may be extreme restlessness on the part of the patient. In such a case one nurse must give her undivided attention to the patient. Padded boards should be fixed to the sides of the bed to prevent him falling out.

*Treatment during convalescence.*—As regards food, the usual *rôle* is to keep the patient on to his fluid diet for ten days after the temperature has fallen to the normal. The nurse's attention must be ever on the watch to ensure this, for as the fever ceases the appetite of the patient may become ravenous. I well remember a case in my student days in which, unknown to the nurse, a friend on the visiting day at the hospital brought a patient an orange. Next day she was seized with peritonitis, with a fatal result. At the postmortem an orange pip was discovered in the peritoneal cavity, together with the perforation through which the pip, and with it her life, had passed. After the period of ten days the diet is cautiously increased under the direction of the doctor. During convalescence the nurse must also be on the look-out for complications, such as phlebitis and bed-sores.

*(To be continued.)*

### The Nurses' Clinic.

### VOMITING.

Sickness, in the sense of vomiting, is one of the commonest ills to which flesh is heir, so much so that like common colds it is often thought lightly of and sometimes not recognised as a possible symptom of disease. Infants in arms frequently vomit after every feed, but if the quantity vomited is small in proportion to the amount taken and the child continues to thrive, it is of no consequence, but is regarded as a kind of safety valve to prevent the stomach being overloaded. If, however, all is not well with the child and he throws up a good deal in a curdled condition with an unpleasant odour, and constant diarrhœa supervenes, it is time to call in a medical man, as these little creatures are rapidly reduced to a state of collapse if not taken in time.

Vomiting in older children may point to the onset of some disease, such as scarlatina or measles, or it may be the result of too rich and unsuitable food, or a sign that worms exist, or a symptom of gastritis, or if apparently quite causeless it may be connected with brain trouble, or if sudden in onset and in combination with abdominal pain and constipation it may point to some internal mischief such as hernia, intussusception, or appendicitis. Sickness in children should always be carefully noted so that a clear report may be given to the doctor. He will want to know the quantity, the appearance, the frequency, the time, the colour, the consistency, whether any pain or griping is associated with it and how the general health and appearance have been affected; unless all these points have been carefully noted by the nurse or mother it is difficult for a busy doctor to elicit really satisfactory answers during the time he is examining the little patient, and children can very seldom render any help in describing their symptoms. Sometimes sickness seems to be of nervous origin and lasts for two or three days with some fever and exhaustion and then goes, but returns again in a few months. These attacks are nevertheless somewhat alarming, and the child should always be seen by a medical man; amateur physicking is as likely to do harm as good.

Vomiting is perhaps even more distressing in an adult, and every nurse knows the importance of noting everything in connection with it. Green "chloroform sickness" following after an operation is very familiar. So long as the patient is vomiting the nurse must be in attendance to keep him as still as possible, instructing him to merely turn his head on one side while she holds the porringer with one hand and with the other gently but firmly supports his wound (particularly if abdominal) to prevent the stitches breaking down under the retching. Simple chloroform sickness calls for no treatment and soon subsides, but any further post-operation vomiting must immediately be reported.

In most cases of cerebral trouble there is sickness to be found, generally associated with headache and having no connection with food. In any accident, too, the chances are that there will be some vomiting, so a little forethought will prevent any distracted rushing about for utensils and clean linen.

Then there is the vomiting of early pregnancy with which some young married women are hardly cognisant. Here the private or district nurse can often soothe an anxious mother's fears by assuring her it is nothing unusual.

Any vomit passed must be kept for the doctor's inspection; in hospitals one does it as a matter of course, but in private houses and in the district how often it happens that well-meaning friends clear away all traces, and perhaps only mention as a casual afterthought that the patient was sick during the day, and no amount of questioning will elicit a satisfactory account of the characteristics of the vomit. It is well to impress upon the friends at the outset the importance of sickness, and with a little tact and persuasion they will soon begin to see it from the nurse's point of view. How varied in colour alone are the different kinds of vomit, so much so that one can hardly expect the lay mind to grasp the significance of even that one point. There is the green sickness of an anæsthetic, the yellow vomit in liver complaints, the dark-coloured hæmatemesis, the brighter red hæmoptysis, the chocolate, viscid vomit associated with liver abscess discharging into the lung, and though, strictly speaking, these two last are sputa, the same nursing rule applies.

The distressing sickness in some stomach affections, such as cancer of the pylorus, is often treated by washing out the stomach, of course, under medical supervision, but a nurse should always be ready to learn lavage, as it is now so frequently employed.

One last word on sea-sickness, for which no definite cure it seems has yet been discovered; in fact, except by the victim it is looked upon as rather a joke, but those who know the intolerable retching, misery, and exhaustion can see little humour in it. A friend going to South Africa was so ill that, in spite of protestations, she insisted on buying a supply of some nostrum warranted to cure sea-sickness in twenty-four hours. This she took liberally, with the result that she was very much worse and practically did not recover the whole voyage. In some cases champagne is excellent, others can take bitter beer, and some crave for pickles or raw tomatoes. In any case never starve before going on board, but eat a good, easily digested meal about two hours before starting.

It has been said that a towel wrung out in hot water and bandaged as tightly round the head as can be borne is an excellent remedy. Others say that a cayenne poultice at the back of the neck is a good recipe; both suggestions might easily be tried as they are safe and simple.

### To Nurses.

We invite contributions from any of our readers, and shall be glad to pay for "Notes on News from the Nursing World," "Incidents in a Nurse's Life," or for articles describing nursing experiences at home or abroad dealing with any nursing question from an original point of view, according to length. The minimum payment is 5s. Contributions on topical subjects are specially welcome. Notices of appointments, letters, entertainments, presentations, and deaths are not paid for, but we are always glad to receive them. All rejected manuscripts are returned in due course, and all payments for manuscripts used are made as early as possible after the beginning of each quarter.

### Incidents in a Nurse's Life.

NEW ZEALAND IN THE 'EIGHTIES.

THE Dunedin Hospital was the last of the important hospitals of New Zealand to make any pretence of training nurses, or even to require previous training in the nurses engaged, and in the 'eighties it was positively benighted in its methods of administration in this respect. An institution of 110 to 120 beds, no female nurse was admitted to the wards set aside for the men-folk. I was the first nurse with three years' English experience to be taken on, and during my two years there I never saw the inside of a men's ward—no more, at least, than might be seen by an occasional and surreptitious glimpse through a door unguardedly left open.

Hearing once that some difficulty was being experienced in procuring a suitable man for a vacancy in one of these departments, I intimated that I would be quite willing to take it myself, either temporarily or otherwise, and it took quite a long time afterwards to reassure the matron of my claim to be considered respectable. She never went into a men's ward herself, the attendants there having a superintendent as uncompromisingly of the masculine gender as themselves. Any domesticated man of good character and active habits did for male nurse; any woman of the same qualifications had hitherto done for a female one; and up to my time the last nurse engaged, albeit she had never seen the inside of a ward before, made her debût as sole night nurse in charge of forty patients scattered over four wards, and including cases of all descriptions of medical and surgical illness, from major operation to chronic rheumatism.

The hours of both day and night nurse were from six to six. Each day-nurse had the care of twenty-two patients—medical in one case, surgical in the other—and in her own department, except for what help she could get out of convalescents, she did absolutely everything herself—looked after her patients, cleaned her fireplace, washed the dishes, cleaned the windows (inside), polished the brasses, scrubbed the tables, lockers, bath-room, lavatory, and ward-kitchen every day, and the whole ward once a week, the latter by degrees, a piece every day as she could find time. And as if this were not enough for one pair of hands, she carried up all the logs required for the ward fire from the basement yard to the top story, and the ward washing was returned rough-dry from the laundry to be folded in the wards. The furniture of the ward-kitchen included a set of flat-irons, so that if a nurse could make time and was "natty" enough she could iron the pillow-cases, but this was not compulsory.

The day-nurse's time off duty was one afternoon a week and every alternate Sunday from two o'clock. The night-nurse had every Sunday evening, coming on at ten instead of six. Consequently the day-nurse, on all day, did an unbroken shift of sixteen hours, with double duty after two. The nurse's dietary was of the simplest description. Butter, tea and sugar, with an occasional tin of jam thrown in, was served to her every week on the ration principle, and kept in a cupboard in her room in readiness for her meals, which, all but her dinner, she took there. As a supplement to these she got for breakfast every day except Sunday—a plate of porridge and a chop, sent up on tin or enamel plates from the main kitchen about half an hour after the ward breakfast. On Sunday liver-and-bacon was substituted for the chop, and scones were provided for tea. Dinner was taken in the matron's company in a room in the basement, containing nothing whatever by way of furniture but a deal table and the requisite number of chairs. The meal was invariably beef or mutton, with potatoes and cabbage; pudding on Wednesdays and Sundays. The nurses lived two in a room (the rooms being adjacent to the wards)—the two day-nurses together, the night-nurse and the housemaid, the latter being the only female servant besides the three laundresses which the institution possessed. She was for attendance on doctor's, dispenser's, secretary's, and matron's rooms; the nurses attended to their own. But in this process there was not much to shift. All the furniture consisted of a bed and a chair, a piece of carpet, and a few hooks in the wall. We improvised tables of inverted packing-cases, draped in white calico—afterwards used for poultice cloth—and we bought our own window-blinds and looking-glass. We went without floor covering, and did not aspire to ornaments, for we neither had time to enjoy nor to dust them.

I had the privilege of seeing a good many changes for the better, however, even in my two years. When I left in 1888 the man-nurse was still in possession of the men's wards, managing them as best he could, considering that he had sixteen patients and neither help nor experience save what he had picked up for himself. But in the female department things had progressed. We had trained nurses, a probationer apiece, and a weekly charwoman. We had also the firewood carried up for us, and the ashes carried down. The stone floor of the dining-room had matting laid down; the packing-cases in our own rooms had given place to real tables, and we had a pudding every day and a change every morning for breakfast. To-day, I believe, in every way the Dunedin Hospital ranks with the best in the colony.

### Maternity Training at the British Lying-in Hospital.

INTERVIEW WITH THE MATRON. BY OUR COMMISSIONER.

A FAIR idea of the urgent needs which are supplied by the British Lying-in Hospital may be gathered from the fact that, as Miss Gertrude Knott, the matron, told me last Thursday afternoon :

"Sister and I booked this morning as many as fifty patients to come in some time during the next three months. This 'booking-day' occurs weekly."

"Their names have to be entered practically three months in advance?"

"Yes, that is indispensable, owing to the great demand for beds."

"And the demand increases?"

"Here are the figures for the last two years. In 1906 the number of in-patients was 515 and the number of out-patients 802; in 1905 they were respectively 484 and 665."

"Can you tell me how these figures compare with the position when you came to Endell Street?"

"When I came here nearly nine years ago, the average number of beds occupied was six; now it is 20. We have 28 beds, and during November and December last year, we had to send several patients home, because we had no room for them. In each case a midwife accompanied them to their home, in order to attend them there. So you can judge from this, that the hospital, though the oldest of its kind in London, has kept pace with the times."

### MATERNITY TRAINING AT THE BRITISH LYING-IN HOSPITAL—*continued.*

### The Patients.

"What is your record in regard to deaths and cases of sepsis?"

"Neither in 1906, nor in 1905, was there either a single maternal death or case of sepsis. We have no resident medical officer here."

"Most of your patients, I suppose, are poor women?"

"The great majority are the very poorest; many of them are the wives of Covent Garden porters. It is a common thing for patients to say that 'the only fortnight of luxury' in their lives is the fortnight they spend here."

"A fortnight is the usual period?"

"Yes, the only exceptions are cases of bad confinements."

"What are the arrangements for your out-door patients?"

"There are three fully-qualified midwives holding the certificate of the Central Midwives Board in attendance on out-door patients attached to the hospital. Each has a very large district. One is in Westminster, another comprises Pentonville, and, in fact, a great part of Islington; and the third Newington and Stamford Hill. They live in their own homes, and work from them. The sister manages the out-patients within a radius of a mile and a half of the hospital, and takes the pupils out to their cases. In these homes the pupils acquire confidence, and learn to be resourceful, especially if a case becomes acute before they send back for help. The out-patients are practically as poor as the others."

### Teaching and Experience.

"How many midwifery pupils and monthly nurses do you train in twelve months?"

"Twenty-six midwifery pupils and about 120 monthly nurses. The average number of applications during the year is 813. We have accommodation for seven pupil midwives and 16 monthly nurses. The former come for four months, and the latter for two, but the pupils have to undergo a preliminary month of training in monthly nursing in the wards before they take their midwifery."

"Do the pupils, after they leave here, go in much for district work?"

"No, I regret to say that most of them at once take up private nursing, because they seem to be afraid of the responsibility of district work. It is a pity, because the experience is so valuable. Nothing, in my opinion, is equal to experience; but, of course, teaching is essential."

"You might give me an idea of the teaching here?"

"The practical teaching in the wards is under the sister and the staff nurses, and I give it in the labour-room. Both sister and myself are constantly in the wards at all hours of the day and night. There are six wards on three floors, with a labour-room between each. A staff nurse is responsible for three wards and for the nursing of the mothers and babies in them."

"What arrangements are made for night duty?"

"Two monthly nurses, and one pupil midwife have been doing night duty so far. As, however, the number of cases is increasing so rapidly, we are going to have a permanent night midwife, who will take all the normal cases."

"You have a visiting staff?"

"Oh, yes, and they deliver lectures to midwifery pupils constantly. I, too, give some. I am afraid that the tendency of the day is to overdo the teaching, and that is why I would emphasise the importance of practical experience and explanation when the demonstrations take place."

### An Ideal Course of Training.

"How many of the monthly nurses and pupils have had general training?"

"About 30 per cent. of both classes, and the percentage is increasing. The age for admission is from twenty-one to forty-five. But I think that it is better to take midwifery training after, and not before general training. My ideal is for a nurse to commence with children's training, go on to general training, and then take midwifery and special training, such as ophthalmic or fever work."

"Have you yourself realised this ideal?"

"Not quite. I had children's training at Pendlebury Hospital, general training at Guy's Hospital, and then I came here as midwifery pupil, and stayed on ever since, first as sister and then as matron. I became matron six years ago. The sister who succeeded me, and is now my valued colleague, was trained at St. Thomas's Hospital, and was afterwards sister at the Royal Waterloo Hospital for Children and Women."

### The Nursery.

"No doubt in recent years many improvements have been effected?"

"When I came there were several flock mattresses, and wooden bedsteads; now there are horse-hair mattresses and Lawson-Tait bedsteads. For bare boards and wooden tables have been substituted linoleum and tile-topped oak tables, and for coloured check quilts, white counterpanes."

"How long has your well-equipped nursery for babies been in existence?"

"Only three years. As you are aware, the old-fashioned plan is to have all the babies washed in the wards with the mothers. The room we have just been into was formerly divided into cubicles. The advantages of the present arrangement are very great. Although the mothers do not have the experience of seeing the babies bathed, the pupils can be more conveniently shown the way than if they were in the wards, and the crying does not disturb the mothers, who are able to sleep from 10.30 to 1. The nurses, too, have more confidence in handling the newly-born, than when the mothers are watching."

### The Home and Hours.

"We abolished the cubicles," continued the matron, "when the Nurses' Home was opened by the Princess of Wales in 1903. It accommodates twenty-two, and contains a separate bedroom for each nurse, separate sitting-rooms for the monthly nurses and the midwifery pupils, and a large dining-hall, in which all the meals are taken."

"The dining-hall is a very fine one."

"It was built on a vacant space, and the earth had to be dug away to make it. With regard to meals, I take the dinner, and the staff nurses the other meals. But we cannot [illegible] so regular as in ordinary hospitals, owing to cases occurring at all times. The fixed hours are: Breakfast at 8, wards at 8.30, dinner at 1.30, tea at 5, supper at 8. The nurses are supposed to be off duty for two hours either morning, afternoon, or evening, but they often give up this time and stay in to see cases."

"What are the terms of admission?"

"The fees for the midwifery course amount to £32, and for monthly nurses £12 14s. 6d. Hospital nurses who go through the midwifery course here are very often offered the appointment of night sister elsewhere subsequently. The value of their training here, without medical students, and in emergencies, is recognised. There is no emergency more trying than a case of hæmorrhage in midwifery. When a nurse is equal to that, she can cope with almost anything that may occur."

### The Indispensable Qualifications.

"How often do you send up pupils to the examinations of the Central Midwives Board?"

"Every four months. The terms have necessarily to overlap a little. The Act has been an advantage to us. We scarcely ever have a failure at the examinations. Midwives should, if possible, be of good education, and we are trying to level up monthly nurses, who, of course, are taken from a lower social class. It is wonderful to see what example and influence will do for them."

"To finish with, I should be glad if you would indicate the qualities which you consider indispensable for maternity nurses?"

"I am sure that no one should take up either midwifery or monthly nursing, who has not an intense love for small babies and can maintain their interest in them. They should possess great patience with them and great sympathy for them. Tact is equally indispensable, and conscientiousness is a vital quality. For those who love the work it is most absorbing, and I cannot possibly exaggerate the importance of nurses impressing upon the mothers the duty of nursing their own infants. If a mother nourish her child for nine months before it is born, she ought to do this on every possible occasion, when the child lives a separate existence."

### "A Probationer Loses Her Sight."

It is an interesting fact, which may be new to some of our readers, though it is doubtless known to others, that the Royal National Pension Fund for Nurses had its origin in the case of a nurse contracting typhoid fever in consequence of a typhoid patient spitting in her mouth. The founder of the Fund was so struck by the cruelty of allowing a zealous nurse to become totally incapacitated for her work owing to her faithful discharge of her duty under most aggravated circumstances that he determined to organise a Nurses' Pension Fund. His aim was to make it possible for all nurses to secure provision for themselves in the event of partial or entire disablement from sickness or accident.

We recall the circumstance because of the sequel to the sad case of a probatioer at the West Ham Poor-law Infirmary, which was referred to in our issue of January 5. It will be remembered that she lost her eyesight through a patient suffering from typhoid fever spitting in her eye, and that all the responsibility the Guardians could take upon themselves to do for her was to assure her that "so long as she behaved herself" she would be employed by them.

The account of the injury inflicted upon the probationer aroused general sympathy, and one of our subscribers in Italy was so obviously touched by the pathetic incident that we have received the following kindly communication with the enclosure mentioned. The letter speaks for itself, and we publish it with much pleasure as a striking instance of the manner in which misfortune incurred in the performance of duty excites just appreciation :—

January 13, 1907.

In The Hospital of January 5 I read a short account of how a probationer at the West Ham Poor-law Infirmary has lost the sight of one eye, while that of the other eye is also impaired. The poor consolation offered by the Guardians induces me to show my sympathy in a practical manner, and I cannot but feel that others might wish to do the same.

I enclose £5 for the unfortunate probationer, and should feel so glad if my small donation could head a list in your paper of other gifts, however small, and so perhaps result in a little "nest-egg" for one who suffers in the discharge of her duty. As I wish to remain anonymous, I shall be glad to read in a future issue of The Hospital that my letter and the note for £5 has reached you safely. Yours faithfully,

"Anonymous."

### A Retrogression in Edinburgh.

With further reference to the letter under this heading which appeared in The Hospital of December 1 last, our correspondent, having been called upon by us for proof of her statements regarding the management of Mavisbank (now New Saughtonhall) Asylum, has failed to substantiate any of these statements. We therefore renew our expression of sincere regret that anything untrue and defamatory concerning Mavisbank (now New Saughtonhall) should have appeared in our columns, and we make an unreserved apology to Sir John Batty Tuke, the Medical Director of that Institution, and to Dr. J. Batty Tuke, the Medical Superintendent, for the publication of the letter in question.

### Everybody's Opinion.

[Correspondence on all subjects is invited, but we cannot in any way be responsible for the opinions expressed by our correspondents. No communication can be entertained if the name and address of the correspondent are not given as a guarantee of good faith, but not necessarily for publication. All correspondents should write on one side of the paper only.]

### MIDWIVES' ASSOCIATION FOR MANCHESTER.

Mrs. Fannie M. Eddie and Mrs. Malcolm, co-secretaries of the Midwives' Association, 9 Albert Square, Manchester, writes : Will you kindly permit us to call the attention of your readers to the Midwives' Association recently formed in Manchester? The objects of the Association are for the benefit and protection of midwives, watching all new legislation affecting the profession and providing legal aid for its members. All registered midwives are qualified for membership. The committee and officers consist of members duly elected by the Association.

[We should like to have a copy of the rules and the names of the committee and officers.—Ed. The Hospital.]

### NURSES' HOME, FAKENHAM.

Miss S. Hamond, Superintendent of the Nurses' Home, Fakenham, writes : I am anxious to bring before the public through the medium of your valuable paper the position in which I find myself with regard to the Fakenham Nurses' Home. This institution was started by my mother, the late Mrs. Robt. Hamond, 33 years ago, with the object of nursing in the cottages, small farmhouses, and in other families where the income is very small, at very reduced fees, and in some cases free of charge. From the commencement of the Home up to April 1905 thousands of cases have been nursed gratuitously and at very reduced fees. I am sure that the public will agree with me that much suffering has been relieved by nurses in the cottages, which could not be touched by any of our hospitals, grand institutions as they are and a glory to our country. Your readers will readily understand that where there are 50 cases that can be removed to hospitals there are hundreds that cannot be moved, and when the breadwinner is laid aside with critical illness the services of a skilled nurse are urgently needed, and an inestimable boon, and the medical men in these days are determined to have them. Sir Alan Manby, the King's physician at Sandringham (who is a valuable member of our Finance Committee), is very strong upon this point, and, as he has a large practice in West Norfolk, his opinion is worth having. Her Majesty Queen Alexandra has been president of the "Nurses' Home" since 1892, and has given an annual subscription of £10 since she has been Queen. I think it possible that there are those interested in nursing who would be sorry to see the work of this institution curtailed, and the carefully chosen hospital-trained nurses, who are able and willing to adapt themselves to all classes, dispersed, for the want of a few hundred pounds. My object in writing is to let the benevolent public know that to carry on the work of the Home we need £1,000 in donations, and that unless some £700 is contributed within a month to meet our present pressing liabilities, this institution, to which I have devoted much of my life, must be seriously crippled. I confidently look to the Christian philanthropists of England to come to my assistance, and thus save the situation.

### WORKHOUSE NURSING.

"A SUPERINTENDENT NURSE" writes: As a superintendent nurse I am truly grateful for the plain speaking of "M. D." on the existing system of workhouse nursing, my position being exactly as "M. D." so ably depicts it. Fully equipped with the necessary credentials, I accepted my present appointment full of enthusiasm for the work, hoping that, by the exercise of tact and goodwill, I might overcome any tendency to unreasonable prejudice. I have failed absolutely. From the beginning a sort of underlying jealousy has been dominant, a spirit of unrest created among the nurses, one of them being told by the master it was quite unnecessary to consult the "sister" about off-duty time, he could always manage it. Sometimes the indisposition of a workhouse inmate has been brought to the notice of a nurse, and her opinion obtained as to the nature of treatment advisable, when I have been on duty. Not only am I in total ignorance of a nurse's credentials when changes occur among the nursing staff, but do not have an opportunity of even speaking to her until she comes to commence her duties. The matron of the workhouse escorts her round the infirmary wards on the day of the appointment; she has also, in the absence of the regular medical officer, escorted his deputy round some of the wards, before making me cognisant of his presence. I could quote many instances of the like indignities I am called upon to cope with. Although associated with Poor-law institutions for a number of years, I never can understand the human weakness at the bottom of this "prejudice." Surely there is sufficient scope in all workhouses for the exercise of that love of power (present with many of us) without encroaching on the corner devoted to the sick. I take it that the aim and ambition of any master and matron should be to prevent overcrowding of the infirmary wards by paying more attention to the cleanliness, clothing, and feeding of the inmates and the children in the schools connected with the workhouse. A great deal of unnecessary work might be spared the often overtaxed superintendent nurse and her staff if separate towels and flannels were provided for each child, the children inspected daily, and in the event of anything infectious or contagious breaking out among them, immediate removal of the patient, stoving of the bed and bedding, cleansing of the bedstead with strong disinfectants, and a free current of air allowed to purify the room. I look forward to a time when a matron and superintendent can work in unison, and thus be a great help to one another.

### A Children's Fancy Dress Dance.

ONE of the prettiest sights and most successful entertainments of the New Year was the children's fancy dress dance at the Hotel Cecil. Its object was charity, but it fulfilled an even more useful purpose by bringing together the children of a large number of zealous workers in the cause of the hospitals, the whole of whom thoroughly enjoyed themselves. Prizes were given for the prettiest costumes selected by the votes of the audience, a system which enables fond parents to gather their friends together and put their child at the head of the poll, whatever costume they may wear. The system is a bad one. If prizes are necessary, judges of knowledge and taste should be selected, and the decision left to them entirely. In any case the ball gave an infinite amount of pleasure to hundreds of children and their parents, and the whole of the arrangements reflect the greatest credit upon Mrs. Johnathan Smith and the small committee of ladies who worked so successfully under her direction.

### "The Hospital" Convalescent Fund.

THE Hon. Secretary begs to acknowledge with thanks the receipt of 2s. 6d. from "Alison," being her annual subscription to this fund.

### Appointments.

ABERGAVENNEY VICTORIA COTTAGE HOSPITAL AND DISPENSARY.—Miss Lillian Smith has been appointed matron. She was trained at the Salford Royal Hospital, Manchester, where she was afterwards sister. As a sister of the Army Nursing Service Reserve, served in South Africa. She has been matron to the Cottage Hospital, Bromyard, and for nearly four years matron to St. Mary's Hospital, Tenbury.

EAST INDIAN RAILWAY COMPANY.—Miss A. I. Thompson has been appointed nurse. She was trained at the Seamen's Hospital, Greenwich. She has since been sister at the Women's Hospital, Soho Square, and charge nurse at the South Eastern Fever Hospital, London. She holds the certificate of the Central Midwives Board.

JOHNSON HOSPITAL, SPALDING.—Miss E. Glenny has been appointed staff nurse. She was trained at Southwark Poor-law Infirmary, East Dulwich.

WEST LONDON HOSPITAL, HAMMERSMITH.—Miss A. McLean has been appointed night superintendent. She was trained at the Royal Infirmary, Liverpool. She has since been sister at the Wolverhampton and Staffordshire General Hospital, sister at the Royal Hospital, Sheffield, and home sister at the Royal Infirmary, Hull.

WIMBLEDON ISOLATION HOSPITAL.—Miss Eliza S. Stone has been appointed charge nurse. She was trained at Lewisham Poor-law Infirmary. She has had four years' experience at the Fountain Fever Hospital, Tooting, London, and has been charge nurse at Leicester Isolation Hospital.

### Queen Victoria's Jubilee Institute.

WE are officially inspired by the authorities of Queen Victoria's Jubilee Institute for Nurses that Miss L. Costelloe has been appointed to Limpsfield, Miss Gertrude Fisher to Sunderland, Miss Emma Gladwin to Willenhall, Miss Harriet Marchant to Warrington, Mrs. Randall to Portslade, Brighton, Miss Emily G. Tindle to Bradford Home, Manchester, and Miss Ethel G. Williams to Widnes. Miss Eva Pashley has been transferred to Holmforth from Lowell Street, Leeds; and Miss E. J. Sutton to Ardwick Green Home, Manchester, from Birmingham.

### Words of Consolation.

### THE ENGLISHMAN'S BIBLE.

IN "The Life and Letters of Frederick W. Faber," by John Edmund Bowden, second edition, p. 337, a most interesting account is given of the effects of Bible reading upon the mind and character of Englishmen. Mr. F. W. Faber became a pervert from Protestantism to Rome, which makes his testimony the more valuable, and accounts for the use of the word "heresy" in the first sentence which follows: "Who will say that the uncommon beauty and marvellous English of the Protestant Bible is not one of the great strongholds of heresy in this country?" "It lives in the ear like a music that can never be forgotten, like the sound of church bells which the convert hardly knows how he can forego. Its felicities seem to be almost things rather than words. It is part of the national mind, and the anchor of the national seriousness. The memory of the dead passes into it; the potent traditions of childhood are stereotyped in its verses. The power of all the griefs and trials of a man is hidden beneath its words. It is the representative of his best moments, and all that has been about him of soft and gentle, and pure and penitent and good, speaks to him out of his English Bible. It is his sacred thing which doubts have never dimmed and controversy never soiled. In the length and breadth of the land there is not a Protestant with one spark of religiousness about him whose spiritual biography is not in his English Bible."

### A Book and its Story.

### SLUM LIFE IN FICTION.*

Annie Holdsworth's new novel gives a realistic picture of London slum life and of the rather amateur attempts of an artist, a young lady singer, and some others at slumming. There is also Lady Dartmoor, the owner of the slum, who, disguised as "Sister Goodfellow," comes and pays angel's visits to that corner of it known as Parsons Rents; and a man, Dunstane Momerie, of humble origin, who has been to the University, run away with his rector's daughter, and as a widower has now a grocer's shop in Parsons Rents. His wife, Priscilla, and their little child, Dollie, have both died through his neglect. Priscilla held strong views on the importance of sheltering from, and elevating the children above, the evil of the cities. Before her death, when, heartbroken by the failure of the ideal which she had imagined her husband capable of reaching, seeing, too late, that he was a mere charlatan and *poseur*, she outlined the scheme of a book to solace her sadness, dealing with city child-life. When dying she left it to her husband to write for her. He has failed to do this. But he allows an elderly lady, Miss Cardew, to write it, and until quite the end of the book, accepts the unqualified praise that it receives, as the author. Miss Cardew has come under the superficial spell of Momerie's good looks. She mistakes his remorse and ungratified vanity for sorrow. He has taken the grocer's shop as a salve to his conscience by living among the poor. Yet, "They say salvation comes through expectation," Dunstane groaned. "He shuddered as he looked round the shop, although his father had been the village grocer, 'Dunstane Momerie, Grocer and General Dealer.' He turned away nauseated. Then he pulled himself together. Bah! there was nothing in common between himself and the grocer down the street. He had not come to Parsons Rents to make a living." Miss Cardew tries to inspire him to write the book of which he is always talking. It is to be a masterpiece. "My friend," she faltered, "I know—I know. You will find consolation for your great sorrow in writing your great book. . . Oh, indeed, you will prevail. To the strong, all things are possible. I see your name already written among the immortals." "Grocer and general dealer," he interrupted. "No, Miss Cardew, I must atone to my lost love by yielding up the place I should have taken among the immortals." Sighing heavily, he waved aside her protest, and passed into the room beyond. There was nothing of the shop there. The low beams of the parlour, the books, pictures, bright chintzes, a lamp swinging on a chain throwing a glow on to a picture in the recess, the portrait of a beautiful sad-faced woman, holding a wan baby. Dunstane's eyes lingered on it. "'Priscilla!' he moaned. He avoided looking at Dollie. He passed to the bureau. 'My future lies coffined there,' he said. The tinkle of the shop bell answered him. He dragged himself scowling to the shop." The picture of his wife and child had been painted by the artist Malden, and named "Our Sister of Pity." Malden was painting the walls of a children's hall to be called "Dollie's Hostel." He is living in Parsons Rents, and a quaint little slum girl, Zo, who dances to organs in the street, comes into Momerie's shop and sees the picture hanging in his sitting-room. "She bounded into the room and stood before the picture of Dunstane's wife and child. Then she curtsied and turned. 'Our Sister of Pity,' she said softly. 'That's what they called her in the hospital where she died. I saw her picture over the bed. The painter, Mr. Malden, put it there. And did you know our Sister too, and the baby Dollie, and her husband, him what broke her heart?' 'It's time you went,' said Dunstane, harshly. 'You're wasting my time.'" Lady Dartmoor, "Sister Goodfellow," as she is known to the inhabitants of Parsons Rents, is described by Zo as being a "tip-top swell, who laughs all the time and don't seem to care what people think of her"; and her socialistic attempts among the children of Parsons Rents Zo considers are a congenial task. "'She enjoys herself, washing the children, and making faces at the dirt. She makes us go to hear her talk of heaven; but there, it's only off and on'—Zo's tone brightened—"and then we have a *real* treat, roast beef and apple pie. Sister Goodfellow has a treat every day till she's sick of it. Then she comes to Parsons Rents for a change.'" Lady Dartmoor invites Zo to a coming-of-age ball given at her town house for Lady Carol. When Zo arrives in a dress sent to her by Lady Dartmoor the policeman on duty will not allow her to pass inside. It all reads as rather impossible. She explains that she has been invited "as a friend of Lady Dartmoor's," and an ironic voice in the crowd calls out, "Let the lydy pass, hofficer. Myke room for the Duchess of Shoreditch." Zo recognises the voice of a certain Jim, an enemy of hers. Then she calls out to the footman, who recognises her as being the dancing girl Lady Dartmoor had had down to her country house to amuse her guests. So she is allowed to pass to the gallery above the Ball-room to look on at the guests dancing. Lady Dartmoor finds her there, and Zo exclaims on seeing her, "'My! It ain't you, Sister Goodfellow? . . . Ain't you going to finish dressing? You'll catch your death of cold like that.' 'Zo, you're too funny! This is full dress. In society, the less you have on the more you're dressed.' 'I didn't know,' said Zo. 'Society must be real brazen.' 'So it is. Real brazen. Clever girl.'" Lady Dartmoor's nephew, Lord Frensham, is in love with her daughter, Lady Carol. He has a cynical face and an ironic manner that belies the sterling qualities which underlie it. Lady Carol has read "The Book of Priscilla," which has taken London by storm, and she is anxious to meet its author. Momerie is invited for a week-end to Penton by Lady Dartmoor. Lady Carol, believing him to have written the book, worships him as a hero. She goes one day to visit some portion of Lord Frensham's estate, and they draw up at a village shop. She sees Momerie's name over the door, and finds it is his birthplace. Inquiries reveal the local reputation of Momerie, the wife of the present proprietor enlightens her on the subject. "'Young Mrs. Momerie were the Rector's daughter, Miss Priscilla. She ran away with young Dunstane, and dearly she paid for it. He broke her heart, which she couldn't expect different, being a grocer, and she used to ladies and gents. . . . Why did she marry him?' 'She had a fine courage, had Miss Priscilla. She thought she could make a man of him. Eh! dear, dear; but the Almighty had give that up before her.'" They drive away, and Lady Carol is very distressed at the disillusionment. From the village they go on to another part of the estate, and Lady Carol sees before her a model village planned on the lines that Priscilla had dreamed of. "'I must be dreaming.' 'No, it is I who have been dreaming, dreaming of bricks and gardens,' said Frensham. 'I thought I'd like a little slum of my own and give the children a chance.' Lady Carol looked at her cousin very humbly. He had not a noble head, no divine love and pity flashed in his eyes. . . . but he had been building homes for the poor while she had scorned him for an idler." The dialogue and characterisation is throughout excellent.

* "The Iron Gates." By Annie Holdsworth. (Fisher Unwin. 6s.)

### Notes and Queries.

**REGULATIONS.**

**The Editor is always willing to answer in this column, without any fee, all reasonable questions, as soon as possible.**

**But the following rules must be carefully observed.**

**1. Every communication must be accompanied by the name and address of the writer.**

**2. The question must always bear upon nursing, directly or indirectly.**

**If an answer is required by letter a fee of half-a-crown must be enclosed with the note containing the inquiry.**

*A Home for Babies.*

(179) I have had maternity training and have had charge of many sick babies, and never lost one, even though sometimes they have been given up by doctors. I should like to open a home for babies. Must I be registered, and where should I apply?—*Baby Lover.*

We strongly advise you not to undertake such an enterprise as you contemplate. These homes are always regarded with suspicion, and trouble frequently ensues.

*Training.*

(180) Can you tell me where I can enter as a probationer without premium; if possible with a small salary? I am 18.—*Shipston.*

We are sorry to disappoint you, but we recommend you not to think of training until you are at least 20 years of age, when there are one or two children's hospitals that might receive you. You had better get "How to Become a Nurse" from The Scientific Press, 28 and 29 Southampton Street, Strand, London, W.C., and study the conditions attending the training of a nurse. You will see that the best age to start training is 23.

*Health Visitors.*

(181) Can you tell me how to set about training as a health visitor?—*Perplexed.*

This title is only used in some provincial districts; but as the qualifications necessary are practically those of sanitary inspectors, it would be advisable for you to train as such. Write for all particulars to the Secretary, National Health Society, 55 Berners Street, W.

*Maternity Nursing.*

(182) I believe there are certain lying-in hospitals where a thorough training in maternity nursing is given in exchange for time instead of premium?—*Harlesden.*

We know of no lying-in hospital which gives training free. You might possibly hear of such a post in an infirmary by advertising, but they are very difficult to obtain.

*Hospital and Poor-law Nursing.*

(183) Can you tell me if, having trained at a general hospital and afterwards acted as sister in a Poor-law infirmary, I am likely to be able to get a good post in a general hospital?—*Wales.*

We think that you will be able to do so. Write first to your training school, and tell the matron what you wish, and very possibly if you have a good record she will take you back into your old hospital again, if there is a vacancy, or help you to get a post elsewhere.

*America.*

(184) *Leah.*—We cannot answer anonymous inquiries. Why not send your name for our information only?

*District Nurse.*

(185) I desire to give a few lectures with practical demonstrations to the village folk, and shall be obliged if you can recommend me a book which will help me in getting up these lectures.—*N. Wales.*

Write for "Lectures on Home Nursing for the Poor," by a District Nurse, which you can procure from The Scientific Press, 28 and 29 Southampton Street, Strand, London, W.C., and is the book you require. Price 1s. 8d. post free.

**Handbooks for Nurses.**

| | Post Free. |
|---|---|
| "A Handbook for Nurses." (Dr. J. K. Watson) ... | 5s. 4d. |
| "How to Become a Nurse." (Sir Henry Burdett) ... | 2s. 4d. |
| "Nurses' Pronouncing Dictionary of Medical Terms" | 2s. 0d. |
| "Art of Massage." (Creighton Hale) ... ... ... | 6s. 0d. |
| "Surgical Bandaging and Dressings." (Johnson Smith.) ... ... ... ... ... ... | 2s. 0d. |
| "Surgical Instruments and Appliances Used in Operations," illustrated. (Harold Burrows, F.R.C.S.) | 1s. 8d. |
| "Hints on Tropical Fevers." (Sister Pollard.) ... | 1s. 8d. |

Of all booksellers or of The Scientific Press, Limited, 28 & 29 Southampton Street, Strand, London, W.C.

### For Reading to the Sick.

O BRING US HOME AT LAST!

O bring us home at last!
Have we much farther through the night to go?
Have we not almost passed
The wilderness? Thou wilt not leave us so.

O bring us home at last!
Our Father! bid our weary wanderings cease;
Uplift the veil, o'ercast
Between our spirits and the home of peace.

*R. T. S.*

All pain, sickness, weariness, distress, languor, agony of mind or body, whether in ourselves or others, is to be treated reverently, seeing in it our Maker's Hand passing over us, fashioning by suffering the imperfect or decayed substance of our souls. Every sorrow is a billow on this world's troublesome sea, which we must pass over on the Cross, to bear us nearer to our home. Each trouble is meant to relax the world's hold over us and our hold upon the world; each loss to make us seek our gain in Heaven.—*E. B. P.*

"Be thou faithful unto death." Perhaps death seems a long way off. Perhaps you say in your heart, "How can I hold out so long?" It may come sooner than you think. Do not let it surprise you. But "if it tarry," if it wait till the threescore years and ten, or even the fourscore years, yet "wait for it"; it will surely come; and when it comes you will be glad that you did wait for it. It is a very great thing to know that there is a fixed limit—fixed in God's counsels, fixed in Christ's foreknowledge—after which all responsibility will be taken off from us, all strain relaxed, all effort ended, every sinew and every nerve resting. Just until death, struggle on; take each day by itself. Try to do the will of God; try to be faithful to Christ, just through that one day; and, in many senses, every evening will be a death and every morning a resurrection. You may say to yourself in the morning, "Let me be faithful, just until the evening"; and at evening you may trust yourself with Christ, just until the morning; and thus, accepting God's merciful ordinance of day and night as it was intended, you will be helped through your pilgrimage. At last that night will come when faithfulness has been consummated; then you will have been "faithful unto death." What then? "I will give thee the crown of life." Is not that, little as we may yet conceive of it, worthy of some endeavour, of some striving, of some self-denial?—*Dean C. Vaughan.*

Surely, yon heaven where angels see God's face,
Is not so distant as we deem
From this low earth? 'Tis but a little space,
The narrow crossing of a slender stream;
'Tis but a veil which winds might blow aside;
Yea, these are all that us of earth divide
From the bright dwelling of the glorified,
The land of which I dream.

*Bonar.*

